# Berberine attenuates diabetic kidney injury via FOXO1/SP1-associated regulation of renal SGLT-2 expression

**DOI:** 10.1186/s13020-026-01533-7

**Published:** 2026-09-29

**Authors:** Jingjing Pan, Xinhe Wang, Xinyuan Han, Hui Cheng, Fengyi Zhang, Yaxin Chen, Changnian Li, Xin Wang, Ziyang Cheng, Yu Zhang, Shuai Zhao, Wenbo Wang, Yahui Li, Yujie Li

**Affiliations:** 1 Shandong University of Traditional Chinese Medicine First Clinical Medical College, Jinan, 250014 People’s Republic of China; 2 Shandong University of Traditional Chinese Medicine College of Traditional Chinese Medicine, Jinan, 250014 People’s Republic of China; 3 Second Affiliated Hospital of Shandong University of Traditional Chinese Medicine, Jinan, 250001 People’s Republic of China

**Keywords:** Berberine, Diabetic kidney injury, FOXO1/SP1/SGLT-2, Renal glucose reabsorption, Phytotherapy

## Abstract

**Background:**

Berberine (BBR), a plant-derived isoquinoline alkaloid, has been reported to exert anti-hyperglycemic and renoprotective effects. However, the mechanism by which BBR regulates renal glucose transport under diabetic conditions remains unclear. Sodium-glucose cotransporter-2 (SGLT-2), mainly expressed in renal proximal tubular epithelial cells, plays a key role in renal glucose reabsorption. This study investigated whether BBR attenuates diabetic kidney injury by regulating SGLT-2 expression and examined the potential involvement of FOXO1 and SP1 in this regulation.

**Methods:**

Db/db mice and high-glucose-exposed human kidney-2 (HK-2) cells were used to evaluate the effects of BBR, empagliflozin (EM), and their combination. Metabolic and renal phenotypes were assessed by biochemical, histological, and ultrastructural analyses. Network pharmacology, public transcriptomic data mining, molecular docking, cellular thermal shift assay (CETSA), quantitative real-time PCR, western blotting, immunofluorescence, nuclear-cytoplasmic fractionation, co-immunoprecipitation, 2-NBDG uptake, dual-luciferase reporter assays, FOXO1 overexpression, and pharmacological interference with SP1-associated regulation were performed to investigate the regulatory mechanism.

**Results:**

Both BBR and EM improved metabolic abnormalities and renal injury in db/db mice, whereas combined treatment produced marked metabolic and renal improvements but was accompanied by an increasing trend in AST. BBR reduced hyperglycemia, improved glucose tolerance and enhanced the glucose-lowering response to exogenous insulin during the ITT, suppressed urinary albumin excretion, and alleviated renal histopathological and ultrastructural damage. Molecular docking and qualitative CETSA findings suggested that FOXO1 may represent a potential molecular target of BBR. In HK-2 cells, high-glucose stimulation increased SGLT-2 expression and glucose uptake, accompanied by dysregulation of FOXO1 and SP1 signaling. BBR downregulated FOXO1 and SGLT-2, restored SP1 and phosphorylated FOXO1 levels, and reduced glucose uptake under high-glucose conditions. FOXO1 overexpression attenuated the BBR-induced reduction in SGLT-2 expression, whereas pharmacological interference with SP1-associated regulation further supported the functional involvement of SP1 in SGLT-2 regulation.

**Conclusion:**

BBR attenuates diabetic kidney injury, at least in part, through FOXO1/SP1-associated regulation of renal SGLT-2 expression. These findings support the involvement of a FOXO1/SP1-associated regulatory axis in renal tubular glucose handling under diabetic conditions.

**Graphical Abstract:**

**Figure:**
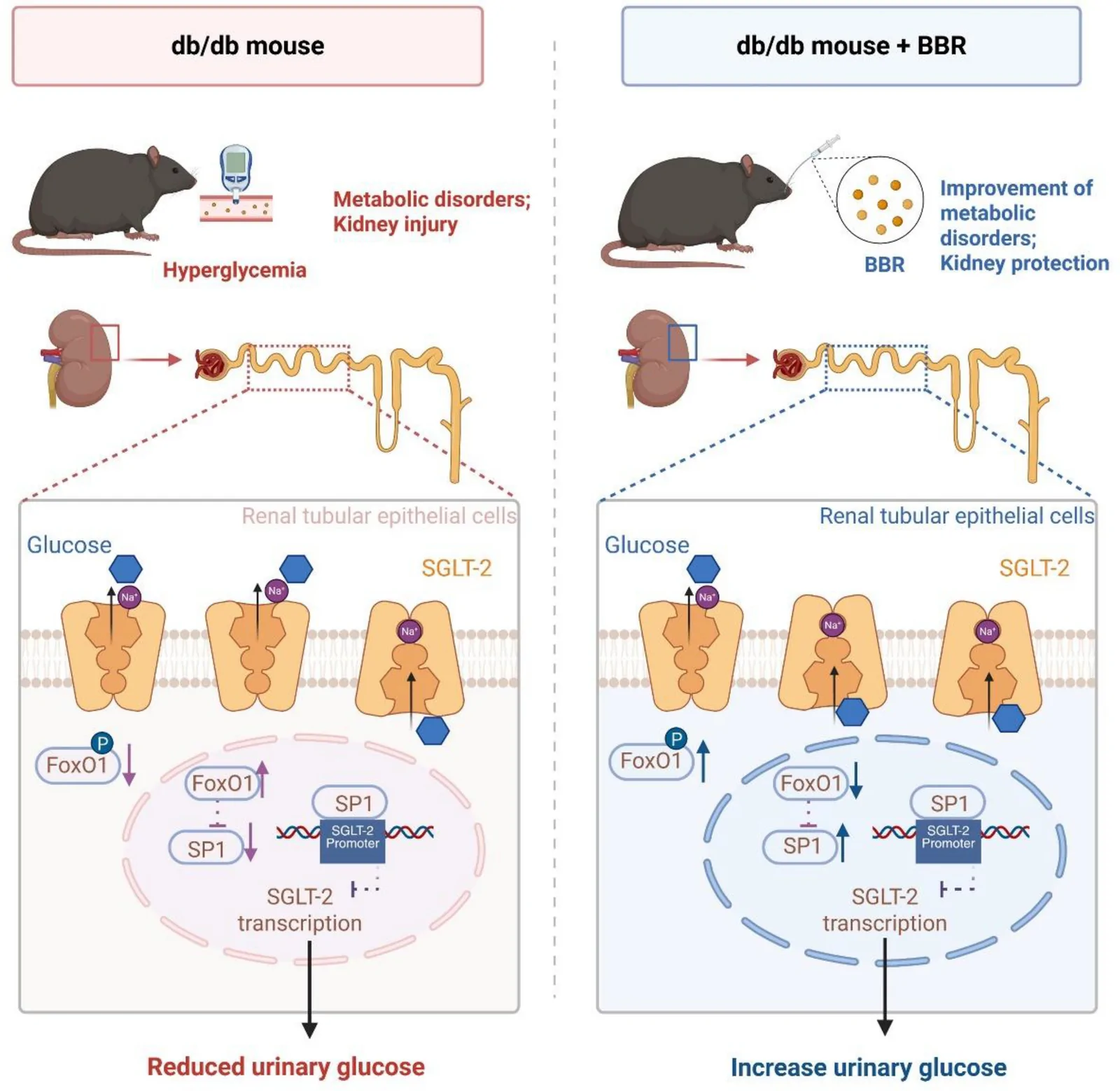

**Supplementary Information:**

The online version contains supplementary material available at https://doi.org/10.1186/s13020-026-01533-7.

## Introduction

Diabetes is a disease with abnormal carbohydrate metabolism. Its main features are reduced glucose utilization and increased gluconeogenesis and glycogenolysis, which lead to hyperglycemia [1]. Diabetes can cause chronic complications in large vessels, small vessels, and the nervous system. It can also lead to acute life-threatening conditions due to metabolic imbalance. Together, these complications substantially impair patients’ quality of life and impose a considerable burden on healthcare systems and society [2]. Over recent decades, the prevalence of diabetes has increased steadily, and it has become an important global public health problem [3, 4]. Therefore, good blood glucose control is important for slowing the progression of metabolic dysfunction and reducing the risk of diabetic complications [5].

At present, sodium-glucose cotransporter 2 (SGLT-2) inhibitors have become an important part of drug treatment for type 2 diabetes [6, 7]. SGLTs are key transporters that mediate glucose reabsorption in renal tubules. SGLT-1 and SGLT-2 are the two main subtypes expressed in renal tubules. Among them, SGLT-2 is mainly located in the S1 and S2 segments of the proximal tubule. It has high capacity and low affinity, and it reabsorbs about 90% of filtered glucose [8]. Therefore, SGLT-2 is considered the core transporter for renal glucose reabsorption [9].

Empagliflozin is a clinically established SGLT-2 inhibitor that lowers blood glucose by reducing proximal tubular glucose reabsorption and promoting urinary glucose excretion. Large clinical outcome trials have demonstrated that empagliflozin reduces cardiovascular events in patients with type 2 diabetes and slows kidney disease progression in patients with type 2 diabetes or chronic kidney disease [10–12]. Moreover, a collaborative meta-analysis of large randomized trials supports the kidney-protective effects of SGLT-2 inhibition across broad patient populations [13]. Therefore, empagliflozin was included in the present study as a pharmacological positive control for SGLT-2-targeted glucose lowering and renoprotection.

Forkhead box O1 (FOXO1) is an important member of the FoxO subfamily in the Forkhead transcription factor family. It plays an essential role in maintaining glucose metabolic homeostasis. Recent findings have demonstrated that FOXO1 is a major transcription factor governing gluconeogenesis in renal proximal tubules. It also plays a role in controlling glucose reabsorption and the transcription of gluconeogenesis-related genes in proximal tubules [14]. As a zinc finger transcription factor family member, specificity protein 1 (SP1) modulates gene transcription by interacting with GC-rich sequences in the promoters of target genes [15]. SP1 has also been reported to alleviate oxidative stress and ferroptosis through the upregulation of Prdx6, thereby attenuating podocyte injury in diabetic nephropathy [16]. In an insulin-induced hepatocyte lipogenesis model, FOXO1 was reported to negatively influence SP1-associated transcriptional regulation [17]. However, whether FOXO1 directly regulates endogenous *SP1* transcription in renal proximal tubular cells remains unclear. Earlier studies indicated that SP1 may participate in the transcriptional regulation of *solute carrier family 5 member 2 (SLC5A2)* in renal proximal tubules. In a primary renal proximal tubule cell model under hypoxic conditions, the expression of SGLT-1/2 and glucose uptake were both significantly reduced. Increased O-GlcNAc modification of SP1 reversed these changes [18]. These data indicate that SP1 may play a critical role in regulating SGLT expression and function in proximal tubules, while also suggesting a potential interaction between FOXO1 and SP1 in the coordinated regulation of glucose metabolism-related target genes. However, under diabetic conditions, there is still no direct evidence showing whether FOXO1, SP1, and SGLT-2 form a continuous regulatory pathway, or whether they are involved in abnormal glucose reabsorption in renal proximal tubules.

Berberine (BBR) is a natural isoquinoline alkaloid widely found in various traditional medicinal plants, particularly *Coptidis Rhizoma* (Huanglian), which has long been used in traditional Chinese medicine for clearing heat, drying dampness, and detoxification [19]. In recent years, BBR has attracted increasing attention because of its diverse pharmacological activities in metabolic diseases, inflammation, liver and kidney dysfunction, and gastrointestinal disorders [20]. Accumulating evidence suggests that BBR regulates glucose metabolism through multiple mechanisms, including improving insulin sensitivity [21], promoting glucose utilization, stimulating glucagon-like peptide−1 (GLP-1) secretion [22], and regulating gut microbiota homeostasis [23]. Meanwhile, studies have demonstrated that BBR has protective effects in many kidney diseases. These effects include reducing oxidative stress [24], inflammation [25], cell death or apoptosis [26, 27], and fibrosis [28]. However, it is still uncertain whether BBR can affect glucose reabsorption in renal proximal tubules under diabetic conditions, and whether it acts through the FOXO1/SP1/SGLT-2 axis.

Therefore, this study aimed to evaluate the effects of BBR on diabetic metabolic abnormalities and renal injury and to investigate the potential involvement of renal SGLT-2 and the FOXO1/SP1-associated regulatory axis.

## Materials and methods

### Reagents and drugs

Berberine (BBR, B21379, purity ≥ 98%) was obtained from Yuanye Biotechnology (Shanghai, China) and dissolved in DMSO (Solarbio, D3870). Empagliflozin was obtained from Boehringer Ingelheim (Germany). HK-2 cells were cultured in DMEM/F-12 medium (Gibco, C11330500BT) supplemented with 10% fetal bovine serum (FBS; Gibco, 10099158). Glucose (Sigma, G8270) and mannitol (Sinopharm, 63008816) were used for cell treatment. A urinary glucose assay kit (Applygen, E1009), urinary protein assay kit (Nanjing Jiancheng, C035-2-1), creatinine assay kit (Nanjing Jiancheng, C011-2-1), and urea nitrogen assay kit (Nanjing Jiancheng, C013-2-1) were used for biochemical measurements. A hematoxylin–eosin staining kit (Beyotime, C0105S) was used for histological staining, and 2-(N-(7-nitrobenz-2-oxa-1,3-diazol-4-yl)amino)−2-deoxyglucose (2-NBDG; Beyotime, S0561S) was used for glucose uptake analysis. RIPA lysis buffer (Servicebio, G20020) and the Nuclear and Cytoplasmic Protein Extraction Kit (Beyotime, P0027) were used for protein extraction. PVDF membranes and ECL substrate were obtained from Millipore. TRIzol reagent (Invitrogen, 15596026), the Evo M-MLV reverse transcription kit (AG11728), and SYBR Green premix (AG11701) were used for RNA extraction, reverse transcription, and quantitative Real-time PCR(qPCR), respectively. Lipofectamine 3000 (Invitrogen) was used for plasmid transfection, and the Dual-Luciferase^®^ Reporter Assay System (Promega) was used for luciferase assays. Mithramycin A (MMA; [Selleck, S8145]), a GC-rich DNA-binding antibiotic that interferes with the binding of SP-family transcription factors to GC-rich promoter elements, was used as a pharmacological tool to interfere with SP1-dependent transcription.

Primary antibodies included phosphorylated Forkhead box O1 (p-FOXO1; Ser256; Cell Signaling Technology, 9461), FOXO1 (HUABIO, ET1608), SP1 (HUABIO, ET1702), SP1 (Proteintech, 66508-1-Ig), SGLT-2 (Immunoway, YT4274), and β-actin (Proteintech, 20536-1-AP). Goat anti-rabbit IgG (Proteintech, RGAR001) and goat anti-mouse IgG (Proteintech, RGAM001) were used as secondary antibodies.

### Network pharmacology

A network pharmacology strategy was used to explore the potential therapeutic targets of berberine against diabetes mellitus. First, berberine-related targets were first retrieved from the Traditional Chinese Medicine Systems Pharmacology Database and Analysis Platform (TCMSP), SwissTargetPrediction, and BATMAN-TCM databases. All predicted targets were merged and duplicates were removed.

Diabetes mellitus-related targets were obtained from the GeneCards and Online Mendelian Inheritance in Man (OMIM) databases using the keyword “diabetes mellitus”, followed by deduplication. The diabetes-related targets were then intersected with the differentially expressed genes (DEGs) derived from the GSE26168 dataset, and the resulting genes were further intersected with the predicted berberine targets to obtain the potential targets of berberine against diabetes. The overlapping targets were visualized using Venn diagrams.

The shared targets were entered into the STRING database (version 11.5) to establish a protein–protein interaction (PPI) network, with the organism limited to Homo sapiens and the minimum required interaction score set at 1.00. The resulting network was subsequently transferred to Cytoscape (version 3.9.1) for graphical presentation and topological evaluation. Within the network, each node corresponded to a protein, whereas each edge denoted an interaction between proteins. Node size and color depth were scaled according to degree values, such that larger and darker nodes reflected higher topological significance. The 20 targets with the highest degree values were defined as hub targets and subjected to further analysis.

### GO functional enrichment and KEGG pathway enrichment analyses

The overlapping targets were subjected to Gene Ontology (GO) enrichment analysis and Kyoto Encyclopedia of Genes and Genomes (KEGG) pathway enrichment analysis using Metascape (https://metascape.org/). GO enrichment analysis was conducted in three categories, including biological process (BP), cellular component (CC), and molecular function (MF). The parameters were set as follows: minimum overlap = 3, P-value cutoff = 0.01, and minimum enrichment = 1.5. After enrichment analysis, the clustering network was exported, and the enriched GO terms and KEGG pathways were ranked according to P values. Finally, bubble plots were generated to display the top 15 GO terms and top 20 KEGG pathways.

### Molecular docking

Molecular docking was carried out to predict the potential interaction between BBR and human FOXO1. The three-dimensional structure of BBR (PubChem CID: 2353) was downloaded from the PubChem database in SDF format and converted into PDB format using Open Babel 3.1.1. The crystal structure of the human FOXO1 DNA-binding domain bound to DBE1 DNA was obtained from the RCSB Protein Data Bank (PDB ID: 3CO6; resolution: 2.10 Å). After receptor and ligand preparation and conversion to PDBQT format, molecular docking was conducted using AutoDock Vina 1.5.7. The search space was centered at x = 9.684, y = −21.629, and z = 15.248 Å, with dimensions of 60 × 60 × 60 Å. The energy range was set to 5 kcal/mol, and 20 docking poses were generated. The pose with the lowest predicted binding energy was selected as the top-ranked docking pose. Predicted contact residues were defined as FOXO1 residues containing at least one heavy atom within 4.0 Å of BBR in the top-ranked pose.

### Analysis of DEGs in GEO dataset

Using the NCBI Gene Expression Omnibus (GEO) database, with “diabetic mellitus” as the search term in Homo sapiens, the GSE26168 dataset was obtained. Using “diabetic kidney disease” as the search term, the GSE30528 dataset was obtained. DEGs were screened via the GEO2R online tool. Samples were classified into control and model groups based on the annotation information in each GEO series, followed by comparison between the two groups. GEO2R performs statistical testing of expression data based on a linear model. It also uses the Benjamini–Hochberg method to adjust *P* values for multiple testing and obtain adjusted *P* values. DEGs were identified using the following thresholds: |log2 fold change (log2FC)|≥ 0.5 and adjusted *P* value < 0.05. At the candidate gene level, we further extracted the expression level of *FOXO1*. Specifically, based on the GEO2R output and the corresponding expression matrix data, we used R Studio software (version 4.2.1) to obtain the expression values of *FOXO1* and *SLC5A2* for further group comparison and visualization.

### Animal model

Male db/db and db/m mice aged 6 weeks and weighing approximately 20 g were purchased from Yantai Jiahe Medical Technology Co., Ltd. All mice were maintained under controlled conditions at 22–24 °C and 55–60% humidity, with a 12 h light/dark cycle and ad libitum access to food and water. All animal procedures were approved by the Animal Ethics Committee of the Affiliated Hospital of Shandong University of Traditional Chinese Medicine (AWE-2023-026) and were performed in compliance with the relevant guidelines for the care and use of laboratory animals.

After a 2-week acclimatization period, 8-week-old db/db mice with fasting blood glucose (FBG) levels exceeding 11.1 mmol/L were randomly assigned to six groups (n = 8 per group): untreated group, empagliflozin group (1 mg/kg), low-, medium-, and high-dose BBR groups (50, 100, and 200 mg/kg, respectively), and a combination group (BBR 100 mg/kg + empagliflozin 1 mg/kg). Empagliflozin was included as a pharmacological positive control for SGLT-2-targeted glucose lowering and renoprotection. The BBR doses and route of administration were selected based on previous studies [21, 29]. All mice were maintained on a standard diet and received the indicated treatments once daily by oral gavage for 16 weeks. The drugs were freshly prepared on the day of administration, and the dosage was adjusted according to changes in body weight.

During the treatment period, fasting blood glucose after 8 h of fasting and body weight were measured every 4 weeks. Urine samples were collected by placing the mice in metabolic cages for 24 h before treatment and at weeks 8 and 16 after treatment initiation. At 24 weeks of age, an oral glucose tolerance test (OGTT) and an insulin tolerance test (ITT) were conducted. The mice were anesthetized with sodium pentobarbital (50 mg/kg, intraperitoneal injection), and blood, kidney, and liver tissue samples were collected. Kidney tissues were either stored at −80 °C for biochemical analysis or fixed in 4% paraformaldehyde for histological examination. Liver tissues were fixed in 4% paraformaldehyde for histological examination.

### Biochemical analysis

Twenty-four-hour urine samples were collected for biochemical analysis. Urinary protein concentration was measured using an enzyme-linked immunosorbent assay (ELISA) kit. Urinary glucose, urinary albumin, urea nitrogen, and creatinine concentrations were determined using the corresponding assay kits. The 24-h excretion of each urinary analyte was calculated by multiplying its measured concentration by the total 24-h urine volume. Serum biochemical indices, such as estimated glomerular filtration rate (eGFR), blood urea nitrogen (BUN), creatinine (CREA), alanine aminotransferase (ALT), aspartate aminotransferase (AST), high-density lipoprotein cholesterol (HDL-C), low-density lipoprotein cholesterol (LDL-C), total cholesterol (CHO), and triglycerides (TG), were determined with an automatic biochemical analyzer.

### Oral glucose tolerance test and insulin tolerance test

For the oral glucose tolerance test (OGTT), mice were fasted for 6 h while allowed free access to water, followed by oral administration of a glucose solution. For the insulin tolerance test (ITT), mice were fasted for 12 h with unrestricted access to water and subsequently received an intraperitoneal injection of insulin (0.5 IU/kg). Blood samples were collected from the tail tip at 0, 30, 60, 90, and 120 min after glucose administration or insulin injection to measure blood glucose levels. Glucose response curves were then generated, and the area under the curve (AUC) was calculated.

### Hematoxylin and eosin staining

Mouse kidney and liver tissues were collected and fixed in 4% paraformaldehyde, embedded in paraffin, and sectioned at a thickness of 5 μm. The sections were then deparaffinized, rehydrated, and stained with hematoxylin and eosin. Kidney sections were used for renal histomorphological evaluation, whereas liver sections were examined for hepatocellular morphology, inflammatory-cell infiltration, and hepatic lobular architecture. Images were acquired using a WISLEAP microscope system.

#### Immunohistochemistry

Mouse kidney sections were processed according to the procedures described above. For antigen retrieval, the sections were heated in 10 mM citrate buffer (pH 6.0) using a microwave and then allowed to cool to room temperature. Endogenous peroxidase activity was quenched with 3% hydrogen peroxide, while nonspecific antibody binding was blocked by treatment with 5% bovine serum albumin (BSA). Subsequently, the sections were incubated with primary antibodies targeting p-FOXO1, FOXO1, SP1, and SGLT-2 overnight at 4 °C. Subsequently, goat anti-rabbit IgG secondary antibody was applied, followed by DAB staining. Images were captured using a WISLEAP microscope, and the proportion of positively stained cells was analyzed using ImageJ.

#### Transmission electron microscopy

Mouse kidney tissues were fixed in 2.5% glutaraldehyde prepared in 0.1 M phosphate buffer (PB, pH 7.4) at 4 °C. The samples were subsequently dehydrated through a graded ethanol series and embedded in resin. Ultrathin sections were then prepared using an ultramicrotome and examined by transmission electron microscopy (TEM) for ultrastructural assessment.

#### Cell culture

HK-2 cells were obtained from the Stem Cell Bank of the Chinese Academy of Sciences (Shanghai, China) and cultured in DMEM/F-12 supplemented with 10% FBS. HK-2 cells were cultured under the above conditions until reaching the appropriate confluence before treatment. The high-glucose group was stimulated with medium containing 50 mM D-glucose for 24 h. The drug treatment groups were treated with 10 μM or 20 μM berberine under the same high-glucose conditions. The osmotic control group was cultured in medium containing 5.5 mM D-glucose plus 44.5 mM mannitol for 24 h to provide an osmotic load equivalent to that of the 50 mM D-glucose condition.

#### 2-NBDG glucose uptake assay

Following high-glucose stimulation and berberine treatment, glucose uptake in HK-2 cells was evaluated using a 2-NBDG fluorescent glucose uptake assay kit. Briefly, 2-NBDG (50 ×) was diluted 1:50 in the Glucose Uptake Assay Buffer provided by the kit to prepare a 1× working solution, which was freshly prepared and protected from light. After the indicated treatments, the culture medium was removed, and the cells were incubated with the 1× 2-NBDG working solution at 37 °C for 30 min. The supernatant was then discarded, and the cells were washed twice with PBS and maintained in PBS before imaging. Green fluorescence images were acquired using a fluorescence microscope, and 2-NBDG fluorescence was detected in the FITC channel under identical acquisition settings for all groups.

#### Nuclear and cytoplasmic protein extraction

Following treatment, nuclear and cytoplasmic proteins from HK-2 cells were isolated using a nuclear/cytoplasmic protein extraction kit according to the manufacturer’s instructions. Briefly, cells were washed with ice-cold PBS, harvested, and lysed in cytoplasmic extraction buffer on ice. After centrifugation, the supernatant was collected as the cytoplasmic protein fraction. The remaining pellet was then resuspended in nuclear extraction buffer, incubated on ice with intermittent vortexing to facilitate complete nuclear lysis, and centrifuged again. The resulting supernatant was collected as the nuclear protein fraction. The cytoplasmic and nuclear protein fractions were used for subsequent Western blot analysis.

#### Western blotting

Total protein was extracted from treated HK-2 cells using RIPA lysis buffer supplemented with protease and phosphatase inhibitors. After centrifugation to remove cell debris, the supernatant was collected, and protein concentration was determined by a conventional method. Equal amounts of protein were mixed with SDS-PAGE loading buffer, denatured, separated by 10% SDS-PAGE, and then transferred onto PVDF membranes. After blocking with 5% BSA at room temperature, the membranes were incubated with primary antibodies against p-FOXO1 (Ser256; Cell Signaling Technology, 9461; 1:1000), FOXO1 (HUABIO, ET1608; 1:1000), SP1 (HUABIO, ET1702; 1:5000), SGLT-2 (Immunoway, YT4274; 1:1000), and β-actin (Proteintech, 20536-1-AP; 1:50000). After washing with TBST, the membranes were exposed to the corresponding HRP-conjugated secondary antibodies (goat anti-rabbit IgG or goat anti-mouse IgG) for 1 h at room temperature. Immunoreactive bands were subsequently visualized using an enhanced chemiluminescence reagent and recorded for analysis.

#### Real-time quantitative PCR

Total RNA was extracted from treated cells using TRIzol reagent. After quantification, RNA was reverse-transcribed into cDNA using the Evo M-MLV reverse transcription kit. Quantitative PCR was then performed on a Bio-Rad CFX real-time PCR system using SYBR Green Premix. The amplification program consisted of an initial denaturation at 95 °C for 30 s, followed by 40 cycles of 95 °C for 5 s and 60 °C for 20 s. β-Actin served as the internal reference, and relative mRNA expression levels were determined using the 2^−ΔΔCt^ method.

#### Immunofluorescence staining

HK-2 cells were cultured on glass coverslips and fixed with 4% paraformaldehyde after treatment, followed by permeabilization with 0.25% Triton X-100 and blocking with 5% BSA for 30 min. Cells were then incubated overnight at 4 °C with primary antibodies against FOXO1 (rabbit, 1:200), SP1 (rabbit, 1:200), SP1 (mouse, 1:50), and SGLT-2 (rabbit, 1:50). Rabbit- and mouse-derived antibodies against SP1 were used for single and double staining. After washing, cells were incubated with the appropriate fluorescent secondary antibodies, counterstained with DAPI, and mounted. Double-staining combinations included FOXO1 + SP1 and SP1 + SGLT-2. Fluorescence images were acquired using a Leica DMi8 microscope under identical acquisition settings for all groups.

#### Co-immunoprecipitation assay

HK-2 cells were lysed in immunoprecipitation lysis buffer supplemented with protease inhibitors. Equal amounts of cellular protein were incubated with an anti-FOXO1 antibody, an anti-SP1 antibody, or the corresponding species-matched control IgG at 4 °C overnight. Protein A/G beads were subsequently added to capture the antibody–protein complexes. After washing, the immunoprecipitated proteins were eluted in loading buffer and analyzed by western blotting using antibodies against FOXO1 and SP1. A portion of each cell lysate was retained as the input control.

#### Plasmid transfection and dual-luciferase reporter assay

HEK-293 T cells were plated in 24-well plates and transfected at suitable confluence with Lipofectamine 3000 in Opti-MEM. The overexpression plasmid and luciferase reporter plasmid were co-transfected at a total DNA amount of approximately 1.50 μg per well, with pRL-SV40 (Promega) used as the Renilla luciferase internal control. After 48 h, cells were harvested and subjected to dual-luciferase reporter assay. Luminescence derived from Firefly and Renilla luciferase was detected sequentially with a luminometer, and promoter activity was evaluated by normalizing firefly luciferase activity to that of renilla luciferase.

#### Lentiviral transduction

FOXO1-overexpressing lentivirus and the corresponding empty vector control lentivirus were used to transduce HK-2 cells. Cells were infected at an MOI of 50 when they reached an appropriate confluence. After further culture, transduction efficiency was evaluated by fluorescence microscopy. Detailed information on the lentiviral vectors and supplier is provided in the Supplementary Materials.

#### Cellular thermal shift assay (CETSA)

HK-2 cells were treated with BBR or DMSO and heated at 37–66 °C for 3 min. Cells were lysed by repeated freeze–thaw cycles between liquid nitrogen and 37 °C, followed by high-speed centrifugation at 4 °C. The soluble fractions were collected and analyzed for FOXO1 expression by Western blot.

#### Statistical analysis

All statistical analyses were carried out using GraphPad Prism 8.0 (San Diego, CA, USA). Experimental data are expressed as the mean ± SEM. The exact sample size for each assay is specified in the corresponding figure legend. For animal experiments, n represents the number of individual mice included in the corresponding analysis. For in vitro experiments, n represents the number of independent biological experiments performed using independently cultured and treated cells. Technical replicate wells and microscopic fields were not considered independent samples; where applicable, values from technical replicates or multiple fields were averaged to generate one value for each biological replicate. Statistical significance between two groups was assessed using Student’s t-test, while comparisons among multiple groups were performed by one-way ANOVA followed by Tukey’s post hoc test. Longitudinal data were analyzed using two-way repeated-measures ANOVA. Differences were considered statistically significant at *P* < 0.05.

## Results

### Berberine improves metabolic abnormalities in db/db mice

**Fig. 1:**
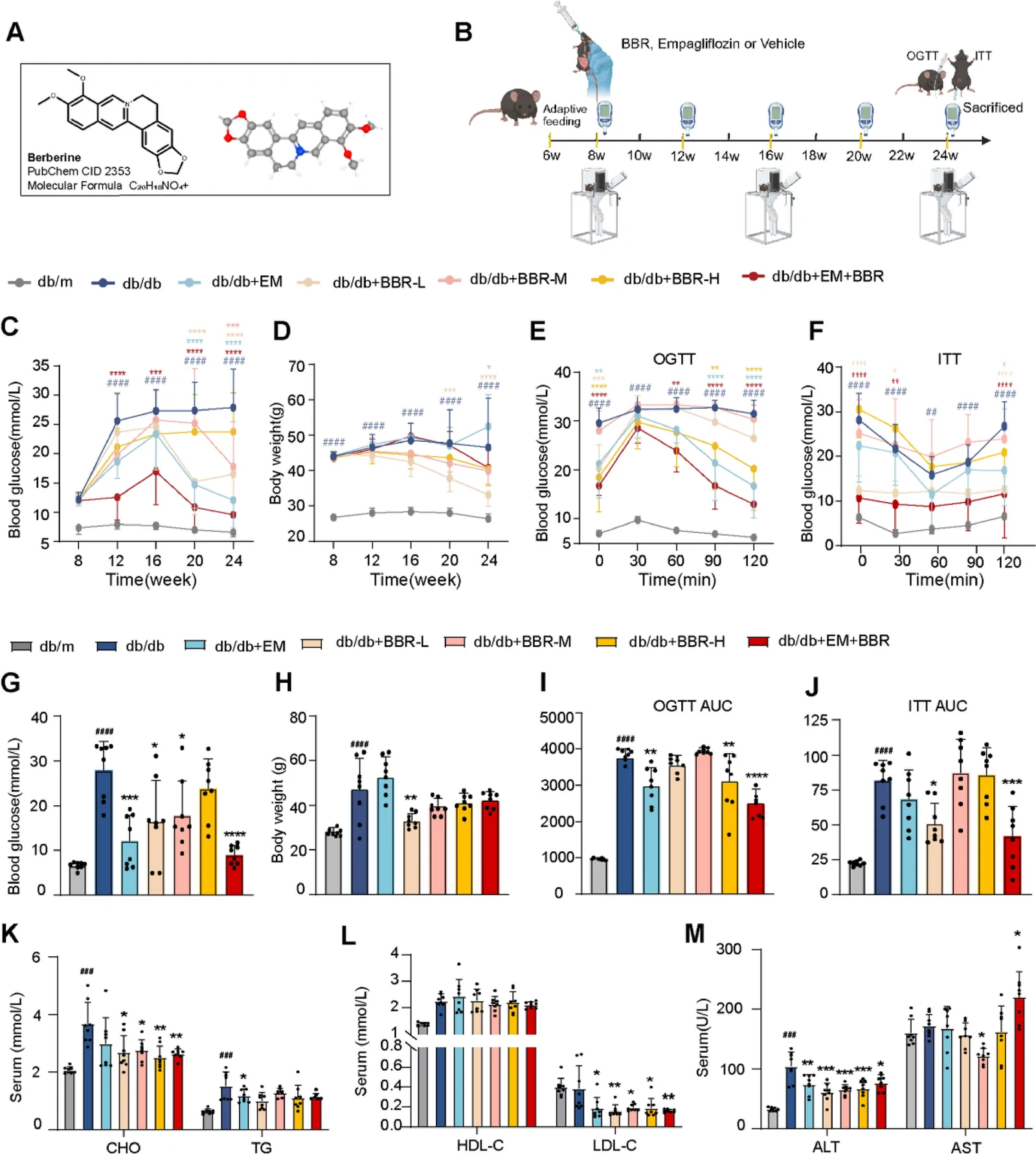
Berberine improves metabolic abnormalities in db/db mice. From 8 to 24 weeks of age, db/db mice received vehicle, EM (1 mg/kg/day), BBR (50, 100, or 200 mg/kg/day), or BBR (100 mg/kg/day) plus EM (1 mg/kg/day) by once-daily oral gavage for 16 weeks. **A** Chemical structure of berberine (BBR). **B** Schematic illustration of the experimental design. db/db mice received vehicle, empagliflozin (EM), BBR, or combined treatment from 8 to 24 weeks of age, followed by metabolic phenotyping before sacrifice. **C**, **D** Dynamic changes in random blood glucose and body weight during the intervention period. **E**, **F** Oral glucose tolerance test (OGTT) and insulin tolerance test (ITT). **G**, **H** Quantification of endpoint random blood glucose and body weight. **I**, **J** Area under the curve (AUC) analysis of OGTT and ITT. **K**, **L** Serum lipid profiles, including total cholesterol (CHO), triglycerides (TG), high-density lipoprotein cholesterol (HDL-C), and low-density lipoprotein cholesterol (LDL-C). **M** Serum alanine aminotransferase (ALT) and aspartate aminotransferase (AST) levels. Data are presented as mean ± SEM (n = 8 mice per group). *P<0.05, **P<0.01, ***P<0.001, ****P<0.0001 vs db/db; ^#^*P*<0.05, ^##^*P*<0.01, ^###^*P*<0.001, ^####^*P*<0.0001 vs db/m

The effects of empagliflozin (EM), berberine (BBR), and their combination on metabolic dysfunction in db/db mice were evaluated (Fig. 1). Figure 1A shows the chemical structure of BBR, and Fig. 1B illustrates the animal experimental design. Compared with db/m controls, db/db mice developed persistent hyperglycemia, progressive body weight gain, impaired glucose tolerance, an attenuated glucose-lowering response to exogenous insulin during the ITT, dyslipidemia, and abnormal liver biochemical indices (Fig. 1C–M).

EM monotherapy markedly lowered random blood glucose but had limited effects on body weight gain (Fig. 1C, D, G, H). EM also improved glucose tolerance and enhanced the glucose-lowering response to exogenous insulin during the ITT, as reflected by reduced OGTT and ITT AUCs (Fig. 1E, F, I, J). EM also partially corrected dyslipidemia and liver injury markers, including decreased TG, LDL-C, and ALT levels (Fig. 1K–M).

The glucose-lowering effect of BBR did not exhibit a monotonic dose–response pattern across the tested doses (Fig. 1C, G). At week 24, mean blood glucose was 16.51 mmol/L (SD, 9.15) in the low-dose BBR group and 23.25 mmol/L (SD, 6.05) in the high-dose BBR group. Although the mean value was lower in the low-dose group, the difference between the low- and high-dose BBR groups was not statistically significant (P = 0.108; Fig. 1G). BBR treatment also attenuated body weight gain to varying degrees (Fig. 1D, H) and partially improved glucose tolerance and the glucose-lowering response to exogenous insulin during the ITT. In addition, BBR ameliorated dyslipidemia, as indicated by reductions in TG and LDL-C, whereas HDL-C did not change significantly (Fig. 1E, F, I–L). ALT was decreased in the BBR-treated groups (Fig. 1M).

Combined EM+BBR treatment also improved several metabolic parameters. The combination reduced random blood glucose and improved glucose tolerance and the glucose-lowering response to exogenous insulin during the ITT, as reflected by the OGTT and ITT AUCs (Fig. 1C, E–J). At week 24, body weight was numerically lower than that in the db/db group, although the difference was not statistically significant (Fig. 1D, H). The combination also decreased CHO, LDL-C, and ALT levels (Fig. 1K–M). However, AST showed an increasing trend in the combination group (Fig. 1M). Liver H&E staining did not reveal overt morphological abnormalities in the BBR+EM group (Supplementary Fig. 2); however, this finding alone cannot exclude mild or subclinical toxicity.

### Berberine ameliorates renal dysfunction and renal histopathological injury in db/db mice

**Fig. 2:**
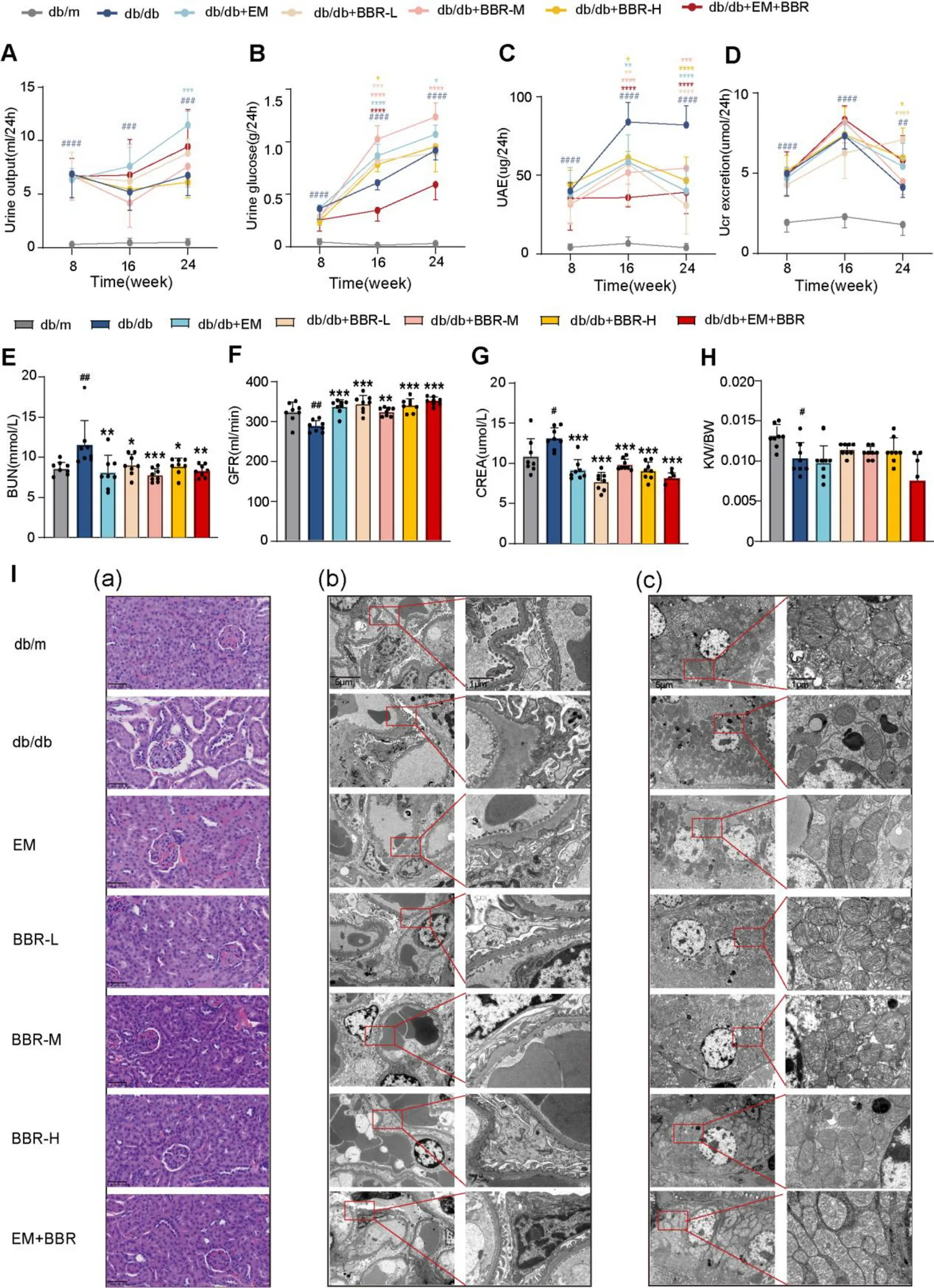
Berberine ameliorates renal dysfunction and renal histopathological injury in db/db mice. From 8 to 24 weeks of age, db/db mice received vehicle, EM (1 mg/kg/day), BBR (50, 100, or 200 mg/kg/day), or BBR (100 mg/kg/day) plus EM (1 mg/kg/day) by once-daily oral gavage for 16 weeks. **A**, **B** Urine volume and urinary glucose excretion in different groups. **C**, **D** Urinary albumin excretion (UAE) and urinary creatinine excretion (Ucr). **E**–**G** Renal biochemical and filtration function parameters, including blood urea nitrogen (BUN), serum creatinine (CREA), and glomerular filtration rate (GFR). **H** Kidney weight/body weight ratio (KW/BW). **I** Representative images of renal histopathological and ultrastructural alterations in each group. **a** Representative H&E staining of renal sections. **b** TEM analysis of glomerular filtration barrier changes. **c** TEM analysis of mitochondrial ultrastructural changes in renal tubular epithelial cells. For urinary and renal biochemical measurements (**A**–**H**), n = 8 mice per group. Representative histological images were obtained from 5 mice per group. Representative TEM images were obtained from n = 3 mice per group, with multiple fields examined for each mouse. Data are presented as mean ± SEM. *P<0.05, **P<0.01, ***P<0.001, ****P<0.0001 vs db/db; ^#^*P*<0.05, ^##^*P*<0.01, ^###^*P*<0.001, ^####^*P*<0.0001 vs db/m

We further evaluated the effects of empagliflozin (EM), berberine (BBR), and their combination on renal injury in db/db mice (Fig. 2). Relative to the db/m group, db/db mice showed higher urine volume, elevated urinary glucose excretion, enhanced urinary albumin excretion (UAE), and altered urinary creatinine excretion (Ucr), together with elevated BUN and serum creatinine levels, decreased GFR, an altered kidney/body weight ratio, and obvious renal histopathological and ultrastructural abnormalities (Fig. 2A–I).

In comparison with the db/db group, the EM-treated group exhibited further increases in urine output and urinary glucose excretion, while UAE and Ucr were improved to some extent (Fig. 2A–D). EM also ameliorated renal biochemical and filtration parameters, as reflected by reduced BUN and serum creatinine and partial restoration of GFR (Fig. 2E–G). H&E staining showed that EM alleviated renal histopathological injury, and TEM further revealed partial improvement in glomerular basement membrane thickening, podocyte foot process effacement, and mitochondrial ultrastructural abnormalities in renal tubular cells (Fig. 2I).

The BBR-treated groups also showed renoprotective effects to varying degrees. Compared with the untreated db/db group, BBR had no apparent effect on urine volume, whereas urinary glucose excretion showed an increasing trend, which was more pronounced in the medium-dose BBR group; meanwhile, BBR reduced UAE and improved Ucr (Fig. 2A–D). BBR treatment also ameliorated renal dysfunction, as indicated by decreased BUN and serum creatinine and improved GFR (Fig. 2E–G). Additionally, the kidney/body weight ratio showed some recovery in certain BBR-treated groups, although the change was not significant (Fig. 2H). H&E staining and TEM further confirmed that BBR attenuated renal structural injury and ultrastructural abnormalities in db/db mice, with the high-dose BBR group showing relatively greater improvement in mitochondrial morphology (Fig. 2I).

The EM+BBR combination group showed improvement in several renal injury-related parameters. Compared with untreated db/db mice, combination treatment did not significantly alter urine output (Fig. 2A). Urinary glucose excretion was significantly reduced at week 16, whereas no significant difference was observed at week 24 (Fig. 2B). UAE was significantly reduced at weeks 16 and 24 (Fig. 2C), while Ucr was not significantly changed by the combination treatment (Fig. 2D). The combination also significantly reduced BUN and serum creatinine and increased GFR (Fig. 2E–G). Representative H&E and TEM images were consistent with attenuation of renal histopathological and ultrastructural abnormalities in the combination group (Fig. 2I).

### Bioinformatic analyses identify *FOXO1* and *SLC5A2* as diabetes-related candidates and broader BBR-associated signaling pathways

**Fig. 3:**
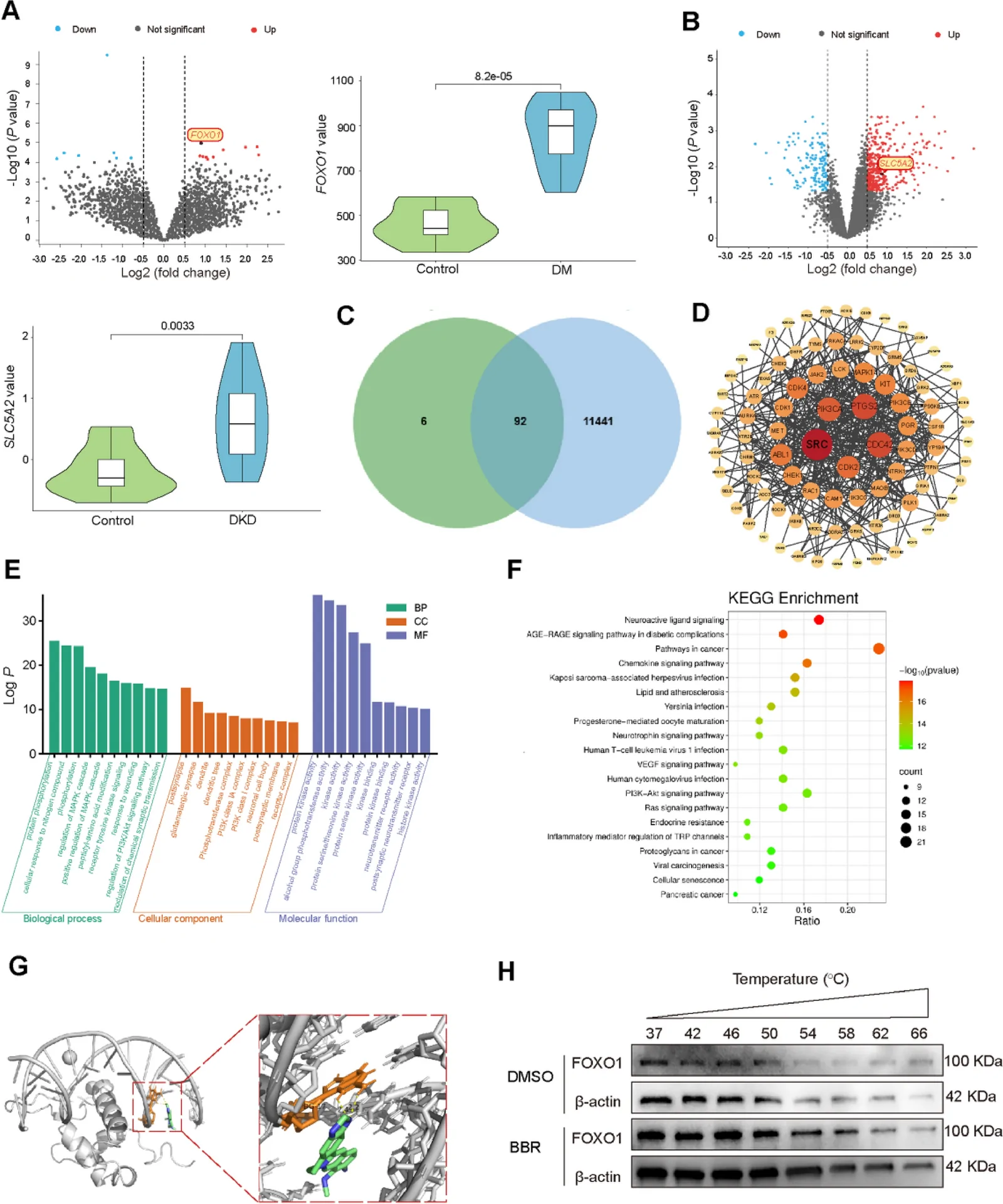
Bioinformatic analyses highlight *FOXO1* and *SLC5A2* as diabetes-related candidate genes and identify putative BBR-associated target networks and signaling pathways. **A** Volcano plot of DEGs in the GSE26168 dataset and expression level of *FOXO1* in different groups. **B** Volcano plot of DEGs in the GSE30528 dataset and expression level of *SLC5A2* in different groups. **C** Venn diagram showing the overlap between predicted targets of BBR and predicted targets related to diabetes. **D** Protein–protein interaction (PPI) network of the common targets. **E** Gene Ontology (GO) enrichment analysis of the common targets. **F** Kyoto Encyclopedia of Genes and Genomes (KEGG) pathway enrichment analysis of the common targets. **G** Molecular docking model showing the binding pattern of BBR with FOXO1. **H** CETSA analysis of FOXO1 thermal stability in HK-2 cells treated with DMSO or BBR. Cells were heated at the indicated temperatures (37–66 °C), and soluble FOXO1 was detected by Western blot

To identify candidate genes and signaling pathways potentially relevant to BBR and diabetes-related renal injury, we performed complementary analyses of public transcriptomic datasets with network pharmacology prediction, functional enrichment analysis, and molecular docking results (Fig. 3). First, DEGs were identified from the GSE26168 dataset, a public dataset related to type 2 diabetes mellitus. Further analysis showed that *FOXO1* was expressed at significantly higher levels in the diabetic group than in the control group (Fig. 3A). This study also analyzed the GSE30528 dataset, which includes gene expression data from glomeruli of patients with diabetic kidney disease and control glomeruli. Since *SLC5A2* encodes SGLT-2, its expression was used to indicate transcriptional changes in SGLT-2. In this dataset, *SLC5A2* was markedly upregulated (Fig. 3B), suggesting that *FOXO1* and SLC5A2 may be involved in diabetes and diabetes-related renal injury. Furthermore, diabetes-related targets retrieved from public databases were combined with the DEGs identified from GSE26168, yielding 11,533 disease-related candidate targets. These candidates were next compared with 98 potential targets of BBR, resulting in 92 overlapping drug–disease targets (Fig. 3C). Protein–protein interaction analysis of these common targets showed relatively close interactions among multiple nodes, with SRC, PTGS2, CDC42, PIK3CA, KIT, and MAPK14 located in the central region of the network (Fig. 3D). GO enrichment analysis indicated that the common targets were mainly involved in hormone responses, regulation of protein kinase activity, MAPK cascade-related processes, and responses to oxygen levels (Fig. 3E). KEGG analysis further revealed significant enrichment in the AGE–RAGE signaling pathway in diabetic complications, PI3K–Akt signaling pathway, and Ras signaling pathway (Fig. 3F). These analyses identified broad diabetes-related signaling contexts but did not directly identify the complete FOXO1/SP1/SGLT-2 regulatory axis. Molecular docking using the human *FOXO1* DNA-binding domain structure (PDB ID: 3CO6) predicted a potential interaction between BBR and *FOXO1* (Fig. 3G). The top-ranked docking pose showed a predicted binding energy of −9.0 kcal/mol. Distance-based contact analysis identified Arg157 and Asn158 as the principal predicted contact residues surrounding BBR. These computational results prioritized *FOXO1* for subsequent experimental evaluation. CETSA analysis (Fig. 3H) showed that soluble FOXO1 generally decreased with increasing temperature. Compared with the DMSO-treated samples, relatively higher levels of soluble FOXO1 were retained in the BBR-treated samples at several elevated temperatures, suggesting that BBR may alter the apparent thermal stability of FOXO1. Collectively, the transcriptomic analyses highlighted *FOXO1* and *SLC5A2* as diabetes-related candidate genes, whereas the network pharmacology and enrichment analyses identified broader putative BBR-associated targets and signaling pathways. SP1 was not identified through the bioinformatic screening but was introduced as a literature-supported candidate mediator based on previous reports suggesting a functional relationship between FOXO1 and SP1 and the involvement of SP1 in renal tubular SGLT regulation. The potential FOXO1–SP1–SGLT-2 regulatory relationship was therefore examined as a hypothesis-driven model in subsequent experiments.

### BBR partially reverses renal FOXO1/SP1/SGLT-2 dysregulation in db/db mice

**Fig. 4:**
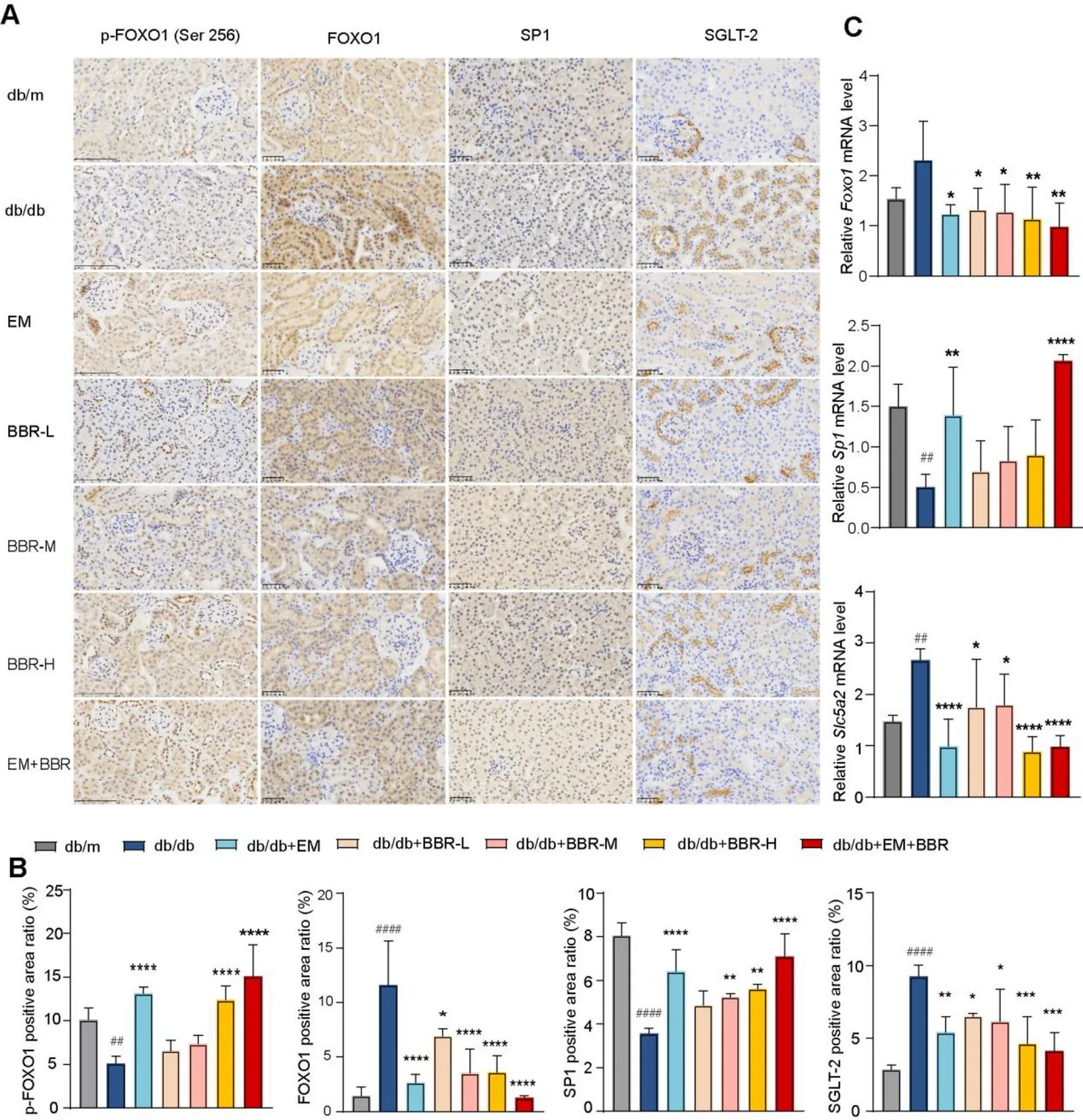
Immunohistochemical and PCR analysis of key target gene expression in renal tissue. From 8 to 24 weeks of age, db/db mice received vehicle, EM (1 mg/kg/day), BBR (50, 100, or 200 mg/kg/day), or BBR (100 mg/kg/day) plus EM (1 mg/kg/day) by once-daily oral gavage for 16 weeks. **A** Representative immunohistochemical staining images of p-FOXO1, FOXO1, SP1, and SGLT-2 in renal tissues from different groups. **B** Quantitative analysis of immunohistochemical staining was performed using kidney sections from n = 5 mice per group. Three nonoverlapping fields were analyzed for each mouse, and the field-level measurements were averaged to obtain one value per mouse. **C** Renal tissue qPCR analysis was performed using n = 3 mice per group. Each biological sample was analyzed in technical triplicate, and the technical replicate values were averaged to obtain one value per mouse. Data are presented as mean ± SEM. *P<0.05, **P<0.01, ***P<0.001, ****P<0.0001 vs db/db; ^#^*P*<0.05, ^##^*P*<0.01, ^###^*P*<0.001, ^####^*P*<0.0001 vs db/m

We further examined changes in FOXO1–SP1–SGLT-2-related molecules in the kidneys of db/db mice using immunohistochemistry and qPCR (Fig. 4). Compared with db/m controls, db/db mice showed markedly reduced p-FOXO1 staining, accompanied by increased total FOXO1 and SGLT-2 immunoreactivity and decreased SP1 immunoreactivity in renal tissue. At the transcriptional level, *Foxo1* mRNA showed an upward trend that did not reach statistical significance, whereas Slc5a2 mRNA was increased and Sp1 mRNA was reduced in db/db mice (Fig. 4A–C).

Compared with the db/db group, EM treatment markedly increased p-FOXO1 staining, reduced total FOXO1 and SGLT-2 immunoreactivity, and partially restored SP1 expression. qPCR analysis further showed that EM significantly reduced Foxo1 and Slc5a2 mRNA levels and increased Sp1 mRNA expression relative to the untreated db/db group (Fig. 4A–C).

The BBR-treated groups also reversed these abnormalities to varying degrees. Immunohistochemical analysis showed that BBR increased p-FOXO1 and SP1 staining while reducing total FOXO1 and SGLT-2 immunoreactivity. The magnitude of these effects varied among doses, with the high-dose BBR group showing a more pronounced increase in p-FOXO1 and suppression of SGLT-2 (Fig. 4A, B). Although Foxo1 mRNA was not significantly elevated in untreated db/db mice relative to db/m controls, BBR treatment significantly reduced Foxo1 mRNA compared with the db/db group. Slc5a2 mRNA was also reduced and Sp1 mRNA showed varying degrees of recovery following BBR treatment (Fig. 4C).

The EM+BBR combination group also showed changes in renal FOXO1-SP1-SGLT-2-related expression. Combination treatment enhanced p-FOXO1 staining, reduced total FOXO1 and SGLT-2 immunoreactivity, and restored SP1 expression. At the transcriptional level, the combination significantly reduced Foxo1 and Slc5a2 mRNA levels and markedly increased Sp1 mRNA expression relative to untreated db/db mice (Fig. 4A–C). Collectively, these findings indicate that EM, BBR, and their combination modulated the renal FOXO1–SP1–SGLT-2 pathway at both the protein and transcriptional levels.

### BBR regulates glucose uptake and the FOXO1/SP1/SGLT-2 axis in model cells

**Fig. 5:**
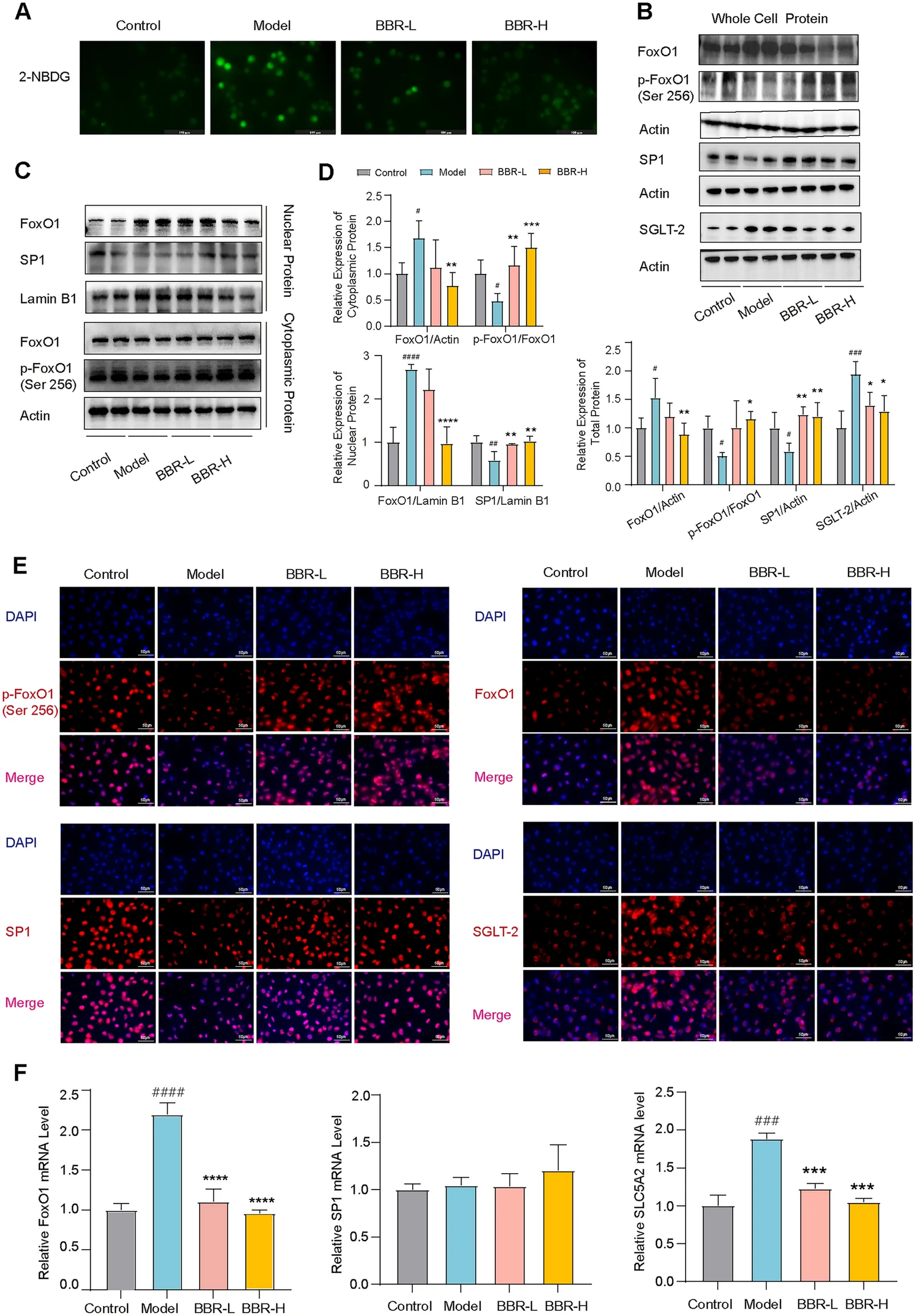
BBR regulates glucose uptake and the FOXO1/SP1/SGLT-2 axis in model cells. HK-2 cells were cultured in medium containing 50 mM D-glucose and treated with BBR (10 or 20 μM) for 24 h. The control group was maintained under an osmotically matched condition (5.5 mM D-glucose plus 44.5 mM mannitol), whereas the high-glucose group was exposed to 50 mM D-glucose. **A** Representative 2-NBDG fluorescence images showing glucose uptake in control, model, and BBR-treated cells. **B** Western blot analysis of FOXO1, p-FOXO1, SP1, and SGLT-2 expression in total cellular protein extracts. **C** Western blot analysis of FOXO1, p-FOXO1, and SP1 expression in nuclear and cytoplasmic fractions. **D** Quantitative analysis of FOXO1, p-FOXO1/FOXO1, and SP1 expression in total, nuclear and cytoplasmic fractions. **E** Representative immunofluorescence staining images of p-FOXO1, FOXO1, SP1, and SGLT-2 in different groups. **F** Relative mRNA expression levels of *FOXO1, SP1*, and *SLC5A2* determined by qPCR. Western blot and densitometric analyses were performed with n = 4 independent biological replicates; the 2-NBDG uptake, immunofluorescence, and qPCR experiments were performed with n = 3 independent biological replicates. Data are presented as the mean ± SEM. *P<0.05, **P<0.01, ***P<0.001 vs model; ^#^*P*<0.05, ^##^*P*<0.01, ^###^*P*<0.001 vs control

To validate the effects of BBR at the cellular level, we performed 2-NBDG uptake assays and analyzed FOXO1–SP1–SGLT-2-related molecules at the protein and transcriptional levels (Fig. 5). Compared with the control group, high glucose-treated cells showed markedly increased 2-NBDG fluorescence, indicating enhanced glucose uptake; this signal was reduced by BBR treatment, with a greater reduction observed in the high-dose group (Fig. 5A). Western blot analysis of total cellular protein showed that high glucose-treated cells exhibited increased FOXO1 and decreased p-FOXO1 expression, accompanied by reduced SP1 and increased SGLT-2 expression. BBR treatment reversed these changes, as reflected by reduced FOXO1, increased p-FOXO1, restored SP1, and suppressed SGLT-2 expression, with a more pronounced effect in the high-dose group (Fig. 5B). Nuclear/cytoplasmic fractionation further showed increased nuclear accumulation of FOXO1 and reduced nuclear SP1 in high glucose-treated cells, together with a decreased cytoplasmic p-FOXO1/FOXO1 ratio. BBR treatment reduced nuclear FOXO1 accumulation, restored nuclear SP1, and increased the cytoplasmic p-FOXO1/FOXO1 ratio (Fig. 5C, D). Immunofluorescence analysis showed changes consistent with these findings, with decreased p-FOXO1 staining and increased FOXO1 and SGLT-2 signals, together with reduced SP1 staining in high glucose-treated cells, all of which were partially reversed by BBR treatment (Fig. 5E). qPCR analysis showed that *FOXO1* and *SLC5A2* mRNA levels were significantly increased in high-glucose-exposed cells and were reduced by BBR treatment, whereas *SP1* mRNA did not differ significantly among the groups (Fig. 5F).

### FOXO1 and SP1 contribute to BBR-mediated regulation of SGLT-2 expression

**Fig. 6:**
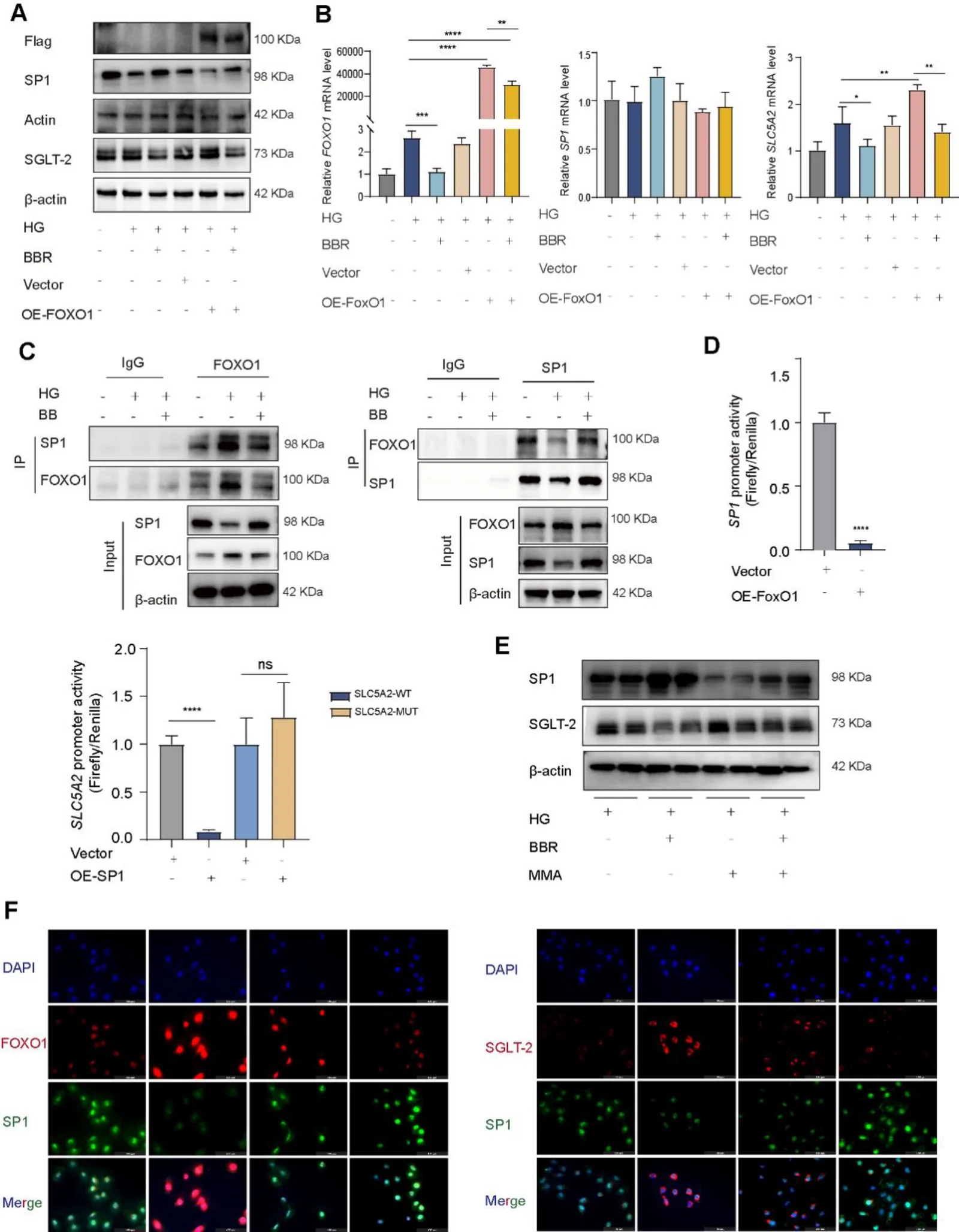
Functional involvement of FOXO1 and SP1 in BBR-mediated regulation of SGLT-2 expression. HK-2 cells were cultured under high-glucose conditions (50 mM D-glucose) and treated with BBR (20 μM) for 24 h. For the indicated experiments, cells were transduced with FOXO1-overexpressing lentivirus at an MOI of 50 or treated with MMA (50 nM) for 24 h. Dual-luciferase reporter activity was measured in HEK-293 T cells 48 h after transfection. **A** Western blot analysis of Flag, SP1, and SGLT-2 expression in HK-2 cells under high-glucose (HG) conditions with FOXO1 overexpression and BBR treatment. **B** Relative mRNA expression levels of *FOXO1*, *SP1*, and *SLC5A2* in the indicated groups, determined by qPCR. **C** Representative reciprocal endogenous Co-IP assays supporting the association between FOXO1 and SP1 in HK-2 cells. Normal IgG served as a negative control. **D** Dual-luciferase promoter-reporter assays showing that FOXO1 overexpression reduced *SP1* promoter-reporter activity, whereas SP1 overexpression reduced wild-type (WT) *SLC5A2* promoter-reporter activity; this effect was no longer observed with the mutant (MUT) reporter. **E** Western blot analysis of SP1 and SGLT-2 expression in HK-2 cells treated with HG, BBR, and mithramycin A (MMA), a pharmacological inhibitor of SP1-dependent transcription. **F** Representative immunofluorescence staining images showing the expression of FOXO1/SP1 and SGLT-2/SP1 in HK-2 cells under the indicated treatment conditions. Nuclei were counterstained with DAPI. qPCR, dual-luciferase reporter, and immunofluorescence experiments were performed with n = 3 independent biological replicates. Data are presented as mean ± SEM. *P<0.05, **P<0.01, ***P<0.001, ****P<0.0001

To evaluate the functional involvement of FOXO1 and SP1 in BBR-mediated regulation of SGLT-2, we performed FOXO1 overexpression experiments in high-glucose-exposed HK-2 cells and dual-luciferase promoter-reporter assays in HEK-293 T cells (Fig. 6). Western blot analysis showed that high glucose (HG) decreased SP1 expression and increased SGLT-2 expression, both of which were reversed by BBR treatment. Under FOXO1 overexpression conditions (OE-FOXO1, Flag-positive), the effects of BBR on SP1 restoration and SGLT-2 suppression were clearly weakened (Fig. 6A).

qPCR analysis showed that HG increased the mRNA levels of *FOXO1* and *SLC5A2*, whereas BBR reduced both. FOXO1 overexpression further increased the mRNA levels of *FOXO1* and *SLC5A2* and weakened the inhibitory effect of BBR on *SLC5A2*. In contrast, *SP1* mRNA did not change significantly among the groups (Fig. 6B).

Reciprocal Co-IP assays were subsequently performed in HK-2 cells. SP1 was detected in FOXO1 immunoprecipitates, whereas FOXO1 was detected in SP1 immunoprecipitates, demonstrating an endogenous association between FOXO1 and SP1 in HK-2 cells (Fig. 6C).

Dual-luciferase promoter-reporter assays in HEK-293 T cells showed that FOXO1 overexpression reduced *SP1* promoter-reporter activity, whereas SP1 overexpression reduced wild-type *SLC5A2* promoter-reporter activity; the latter effect was no longer observed after mutation of the predicted SP1-binding site (Fig. 6D).

In addition, mithramycin A (MMA) markedly weakened the BBR-induced increase in SP1 and decrease in SGLT-2 under HG conditions (Fig. 6E). Dual immunofluorescence staining showed increased FOXO1 and SGLT-2 signals and decreased SP1 in the model group, which were partially reversed by BBR treatment (Fig. 6F). Together, these findings support the functional involvement of FOXO1 and SP1 in BBR-mediated regulation of SGLT-2 expression.

## Discussion

Tubular glucose reabsorption plays an important role in maintaining hyperglycemia in diabetes. Therefore, inhibiting SGLT-2-mediated glucose reabsorption and promoting urinary glucose excretion is an effective strategy for improving hyperglycemia [8, 30, 31]. In recent years, natural products have received increasing attention because they act through multiple targets and pathways. In this study, we showed that BBR improved the diabetic metabolic phenotype and protected renal function. In addition, the results suggest that BBR may inhibit the increase in glucose reabsorption in renal tubules and promote urinary glucose excretion by regulating the FOXO1/SP1/SGLT-2 axis. Empagliflozin was included as a pharmacological positive control because its SGLT-2-inhibitory, glucosuric, and renoprotective effects are clinically established [10–13]. It provided a benchmark for evaluating the effects of BBR on renal glucose handling and kidney injury, rather than serving as a mechanistic positive control for the FOXO1/SP1 regulatory axis.

In this study, db/db mice were used to evaluate the metabolic and renal effects of BBR and EM. Consistent with previous reports, db/db mice exhibited marked hyperglycemia, progressive body-weight gain, impaired glucose tolerance, an attenuated glucose-lowering response to exogenous insulin during the ITT, dyslipidemia, and elevated serum ALT levels. BBR treatment improved these metabolic abnormalities to varying degrees, including reductions in blood glucose and body-weight gain, partial improvement in glucose tolerance, an enhanced glucose-lowering response to exogenous insulin during the ITT, and decreases in TG, LDL-C, and ALT levels, in agreement with previous experimental and clinical findings [32–34]. Notably, however, the glucose-lowering effect of BBR did not follow a strictly monotonic dose–response pattern. Notably, the glucose-lowering effect of BBR did not follow a monotonic dose–response pattern. At week 24, mean blood glucose was numerically lower in the low-dose than in the high-dose BBR group, although the difference was not statistically significant. The magnitude of changes in body weight and ITT AUC also varied across the BBR doses. This apparent dissociation may reflect the multitarget and endpoint-dependent metabolic actions of BBR rather than a simple dose-dependent consequence of renal SGLT-2 modulation. Blood glucose represents an integrated systemic endpoint influenced by body-weight changes, insulin responsiveness, hepatic glucose production, intestinal glucose handling, and gut microbiota-associated metabolism. Previous clinical and experimental studies further support the multitarget metabolic actions of BBR. In the PREMOTE randomized clinical trial, the glucose-lowering effects of BBR were associated with remodeling of the gut microbiome and microbial bile acid metabolism [23], whereas another study identified KCNH6-dependent glucose-stimulated insulin secretion as an additional mechanism of BBR action [35]. These findings provide a broader context for the non-monotonic glucose-lowering response observed in the present study and support interpreting BBR as a multitarget metabolic regulator rather than a selective SGLT-2 inhibitor. Therefore, the systemic glucose-lowering effect of BBR should not be attributed exclusively to its action on renal SGLT-2, and the dose associated with the greatest effect may differ among metabolic and renal endpoints. Moreover, because ITT AUC may be affected by between-group differences in baseline glucose levels, the ITT findings should be interpreted as supportive evidence of improved overall insulin responsiveness rather than direct proof that low-dose BBR exerted the strongest insulin-sensitizing effect. At the renal level, BBR improved both functional and structural manifestations of diabetic kidney injury and promoted urinary glucose excretion. Combined treatment with BBR and EM improved several metabolic and renal parameters. However, AST showed an increasing trend in the combination group, raising a potential safety concern. Liver H&E staining did not reveal obvious hepatocellular necrosis, marked inflammatory-cell infiltration, or severe disruption of hepatic lobular architecture. Nevertheless, the absence of overt histopathological abnormalities does not exclude mild, focal, transient, or subcellular hepatic stress. Furthermore, AST is not liver-specific and may also be influenced by extrahepatic tissues. Because additional hepatic and extrahepatic safety markers, such as bilirubin, alkaline phosphatase, gamma-glutamyl transferase, creatine kinase, and lactate dehydrogenase, were not evaluated, the source and significance of the AST change remain uncertain. Accordingly, the present data are insufficient to establish a therapeutic advantage of the combined regimen over either monotherapy, and additional safety assessment is required before its translational potential can be adequately evaluated.

Previous studies have attributed the renoprotective effects of BBR to multiple mechanisms, including attenuation of renal inflammation and extracellular matrix accumulation, preservation of podocyte homeostasis, and improvement of mitochondrial energy metabolism and fatty acid oxidation [25, 26, 36–40]. A systematic review and meta-analysis further supports the overall renoprotective effects of BBR in diabetic kidney disease [41]. These findings indicate that BBR may exert protective effects at both glomerular and tubular levels. The present study extends this literature by focusing on proximal tubular glucose handling and renal SGLT-2 expression. Thus, altered SGLT-2-associated glucose reabsorption may represent an additional component of BBR-related renoprotection rather than its exclusive mechanism. SGLT-2 is mainly expressed in proximal tubular epithelial cells and mediates the reabsorption of most filtered glucose and part of sodium. Under diabetic conditions, increased SGLT-2 activity reduces sodium delivery to the macula densa and weakens tubuloglomerular feedback, leading to afferent arteriolar dilation, increased intraglomerular pressure, and aggravated hyperfiltration. This further increases the metabolic burden on renal tubules and promotes the progression of diabetic kidney injury [42–46]. In the present study, BBR significantly reduced SGLT-2 expression in renal tissues of db/db mice and in high glucose-induced HK-2 cells, accompanied by increased urinary glucose excretion in db/db mice and decreased glucose uptake in HK-2 cells. These results suggest that BBR may exert a protective effect against diabetic kidney injury by inhibiting SGLT-2-mediated glucose reabsorption.

To explore the molecular basis underlying the regulation of SGLT-2 by BBR, we performed complementary bioinformatic analyses. However, the FOXO1/SP1/SGLT-2 axis was not directly identified as a complete module by the network pharmacology analysis. The PPI hub targets and enriched pathways instead provided a broader diabetes-related signaling context, including the PI3K–Akt, AGE–RAGE, MAPK, and Ras pathways. Public transcriptomic analyses independently highlighted *FOXO1* and *SLC5A2* as diabetes- or diabetic kidney disease-related candidate genes. SP1 was not identified by the bioinformatic screening but was introduced as a literature-supported candidate mediator based on previous studies suggesting a functional relationship between FOXO1 and SP1 and the involvement of SP1 in renal tubular SGLT regulation. Therefore, the bioinformatic analyses should be interpreted as a hypothesis-generating step rather than definitive evidence for the complete FOXO1/SP1/SGLT-2 axis. The functional involvement of FOXO1 and SP1 in SGLT-2 regulation was subsequently examined through renal tissue analyses, HK-2 cell experiments, FOXO1 overexpression, pharmacological interference with SP1-dependent transcription, and dual-luciferase reporter assays.

**Fig. 7:**
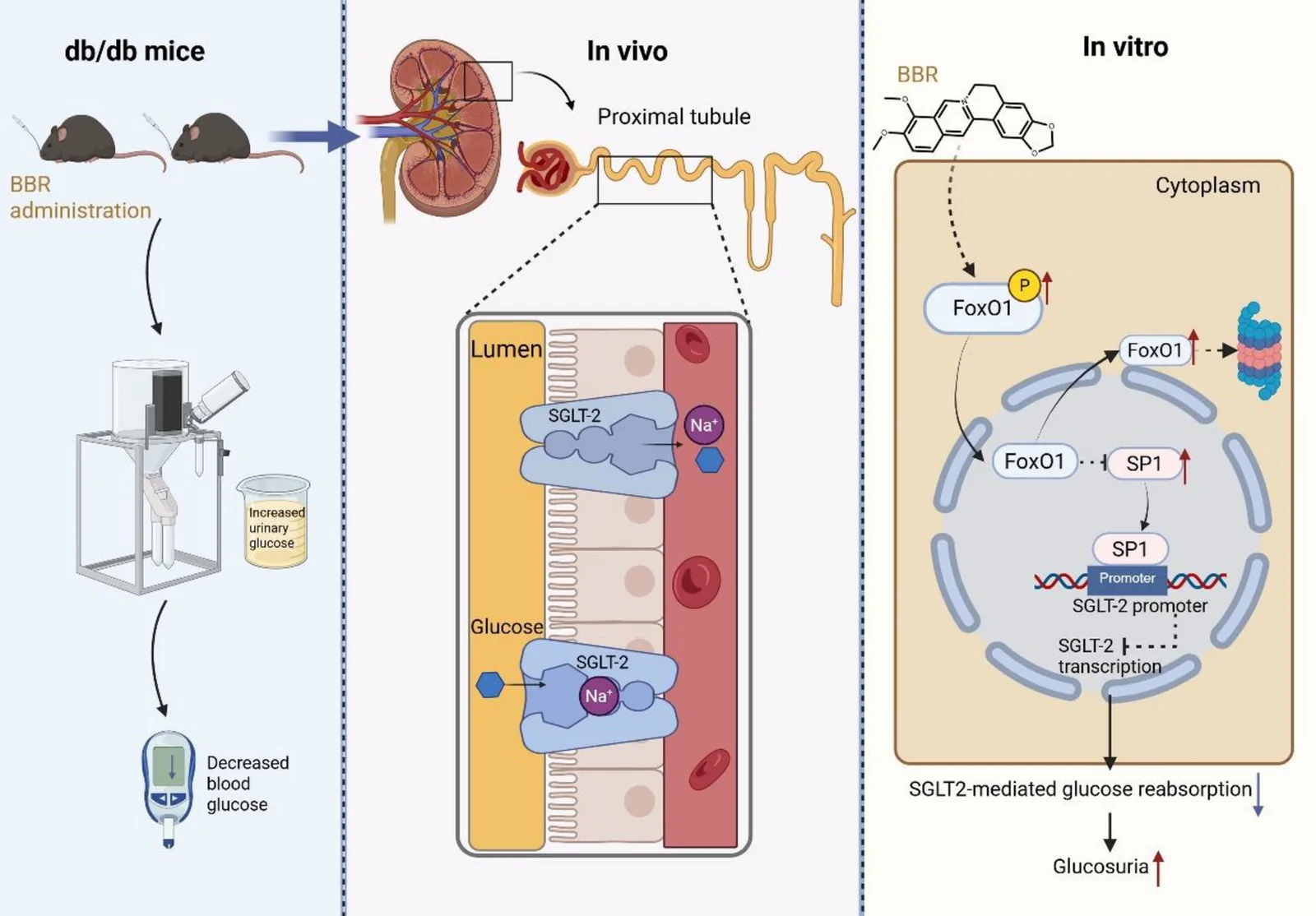
Proposed model of BBR-mediated regulation of the FOXO1/SP1/SGLT-2 axis in diabetic kidney injury. BBR reduced blood glucose levels and increased urinary glucose excretion in db/db mice, accompanied by reduced renal SGLT-2 expression and attenuation of diabetic kidney injury. In high-glucose-exposed HK-2 cells, BBR increased FOXO1 Ser256 phosphorylation, reduced nuclear FOXO1 accumulation, restored SP1 protein expression, decreased SGLT-2 expression, and reduced cellular glucose uptake. Collectively, these findings support a proposed FOXO1/SP1-associated regulatory mechanism linking BBR treatment to altered SGLT-2 expression and renal tubular glucose handling

Molecular docking and CETSA provided complementary evidence consistent with cellular target engagement of FOXO1 by BBR, supporting FOXO1 as a candidate proximal cellular target. Previous studies have shown that abnormal regulation of transcription factors plays an important role in the onset and progression of diabetic kidney injury. Among these factors, abnormal regulation of FOXO1 is closely associated with podocyte injury, tubulointerstitial injury, and fibrosis [47, 48]. Meanwhile, SP1 is a transcription factor that mediates the transcriptional regulation of multiple metabolism-related genes. Its expression and activity are influenced by the pathological microenvironment, and it participates in renal pathological processes, particularly renal fibrosis and renal epithelial cell apoptosis [49–51]. Consistent with this background, total FOXO1 expression was increased, whereas p-FOXO1 expression was decreased, accompanied by decreased SP1 protein expression and increased SGLT-2 expression. BBR treatment partially reversed these changes, suggesting that diabetic conditions are associated with changes in both FOXO1 abundance and phosphorylation status. Notably, FOXO1 overexpression attenuated the BBR-induced restoration of SP1 protein expression and suppression of SGLT-2 expression in high-glucose-exposed HK-2 cells. Dual-luciferase promoter-reporter assays showed that FOXO1 overexpression reduced *SP1* promoter-reporter activity, whereas SP1 overexpression reduced wild-type *SLC5A2* promoter-reporter activity; the latter effect was no longer observed after mutation of the predicted SP1-binding site. Pharmacological interference with SP1-dependent transcription attenuated the BBR-induced increase in SP1 protein and decrease in SGLT-2 expression, further supporting the functional involvement of SP1. Taken together, these findings support the involvement of a FOXO1/SP1-associated regulatory axis in BBR-mediated regulation of SGLT-2 expression under diabetic conditions, which may contribute to the renoprotective effects of BBR (Fig. 7).

Although the dual-luciferase promoter-reporter assays showed that FOXO1 and SP1 altered *SP1* and *SLC5A2* promoter-reporter activity, respectively, these assays were conducted in HEK-293 T cells. Therefore, the findings reflect promoter-regulatory potential in an exogenous reporter system but do not establish endogenous promoter occupancy in renal tubular epithelial cells. Reciprocal Co-IP assays supported an endogenous association between FOXO1 and SP1 in HK-2 cells, providing additional evidence for their functional relationship. Thus, the present findings support a FOXO1/SP1-associated regulatory axis but do not prove a direct chromatin-binding cascade. ChIP-qPCR will be required to determine whether FOXO1 occupies the endogenous *SP1* promoter and whether SP1 occupies the *SLC5A2* promoter in renal tubular cells.

Notably, total FOXO1 protein was increased, whereas p-FOXO1 at Ser256 was decreased, under diabetic and high-glucose conditions. These changes are not necessarily contradictory. The p-FOXO1 antibody used in this study recognizes Ser256, an Akt-regulated inhibitory phosphorylation site. Phosphorylation at this residue suppresses FOXO1 transcriptional activity by promoting its nuclear exclusion and affecting its DNA-binding capacity, and may also facilitate phosphorylation-dependent proteasomal degradation [52, 53]. Consistent with this mechanism, nuclear–cytoplasmic fractionation showed increased nuclear accumulation of FOXO1 in high-glucose-exposed HK-2 cells. Therefore, the combination of increased total FOXO1, reduced Ser256 phosphorylation, and enhanced nuclear FOXO1 accumulation is more consistent with reduced inhibitory phosphorylation and increased nuclear retention of FOXO1 than with suppression of FOXO1 activity. *Foxo1* mRNA showed only a nonsignificant upward trend in the untreated model groups, indicating that the increase in total FOXO1 protein cannot be confidently attributed to compensatory transcriptional upregulation. Altered protein stability or turnover may also contribute to FOXO1 protein accumulation, although this possibility was not directly examined. Notably, BBR significantly reduced *FOXO1* mRNA and reversed the changes in p-FOXO1 and nuclear accumulation, suggesting that BBR may regulate FOXO1 at both the transcriptional and post-translational levels. The biological effects of FOXO1 are highly context-dependent and may vary according to cell type, disease stage, phosphorylation status, subcellular localization, downstream target genes, and the pathological process examined [54, 55]. Previous studies have shown that FOXO1 can attenuate diabetic tubulointerstitial fibrosis and tubular apoptosis through inhibition of STAT1 signaling [47]. In contrast, the present study focused on proximal tubular glucose handling and SP1-associated regulation of *SLC5A2* promoter activity and SGLT-2 expression. Thus, our findings do not exclude the previously reported protective FOXO1/STAT1 mechanism, but suggest that FOXO1 may exert different effects through distinct downstream transcriptional programs.

In renal tissues from db/db mice, *Sp1* mRNA and protein changed in broadly similar directions. In contrast, SP1 protein abundance and nuclear localization changed markedly in high-glucose-exposed HK-2 cells, whereas *SP1* mRNA remained unchanged. This discrepancy indicates that the observed changes in SP1 protein abundance cannot be explained solely by changes in *SP1* mRNA levels. However, the underlying mechanism remains unresolved because SP1 protein stability, turnover, and post-translational regulation were not directly examined in the present study. Therefore, the FOXO1/SP1/SGLT-2 pathway should be regarded as a functionally supported regulatory axis rather than a simple linear transcriptional cascade.

Several limitations should be acknowledged. First, this study mainly used the db/db mouse model, which represents obesity-associated type 2 diabetes and may not fully capture other diabetic conditions or disease stages. Second, fasting insulin was not measured, precluding calculation of fasting-based surrogate indices of insulin resistance and limiting assessment of basal insulin status. Although the ITT evaluated the whole-body glucose-lowering response to exogenous insulin, it could not substitute for direct measurements of insulin sensitivity. Third, the precise regulatory relationships within the FOXO1–SP1–SGLT-2 axis remain incompletely defined. The reporter assays were performed in HEK-293 T cells, Co-IP could not distinguish direct from indirect protein association, and promoter occupancy was not examined by ChIP-qPCR. Moreover, the discordance between SP1 mRNA and protein levels remains unresolved, as SP1 protein stability was not directly assessed. Future cycloheximide-chase experiments are needed to clarify this discrepancy. Fourth, the safety of combined BBR and EM treatment requires additional evaluation. Although no overt hepatic histopathological abnormalities were observed, AST showed an increasing trend, and only one dose combination was tested. Further studies using additional diabetic models, direct metabolic assessments, chromatin-based mechanistic analyses, and broader dose-dependent safety evaluations are warranted.

## Conclusion

In conclusion, BBR improved metabolic abnormalities, renal function, and renal structural damage in diabetic models. These effects were accompanied by reduced renal SGLT-2 expression, increased urinary glucose excretion, and changes in FOXO1 and SP1 expression. Collectively, the findings support the possible involvement of the FOXO1/SP1/SGLT-2 regulatory axis in BBR-mediated regulation of renal tubular glucose handling and amelioration of diabetic kidney injury.

## Supplementary Information

Supplementary Material 1

## Data Availability

The datasets analyzed in the current study are available in the GEO repository at the following accession links: https://www.ncbi.nlm.nih.gov/geo/query/acc.cgi?acc=GSE30528 and https://www.ncbi.nlm.nih.gov/geo/query/acc.cgi?acc=GSE26168.

## Abbreviations

2-NBDG: 2-(N-(7-nitrobenz-2-oxa-1,3-diazol-4-yl)amino)-2-deoxyglucose
ALT: Alanine aminotransferase
AST: Aspartate aminotransferase
AUC: Area under the curve
BBR: Berberine
BUN: Blood urea nitrogen
CHO: Total cholesterol
CREA: Creatinine
eGFR: Estimated glomerular filtration rate
ELISA: Enzyme-linked immunosorbent assay
FBG: Fasting blood glucose
FOXO1: Forkhead box O1
GEO: Gene Expression Omnibus
GLP-1: Glucagon-like peptide-1
HDL: High-density lipoprotein
HK-2: Human kidney-2
ITT: Insulin tolerance test
LDL: Low-density lipoprotein
MMA: Mithramycin A
OGTT: Oral glucose tolerance test
p-FOXO1: Phosphorylated Forkhead box O1
SGLT-2: Sodium-glucose cotransporter 2
*SLC5A2*: Solute carrier family 5 member 2
SP1: Specificity protein 1
TEM: Transmission electron microscopy
TG: Triglycerides
UAE: Urinary albumin excretion
Ucr: Urinary creatinine excretion
