# Supplementary material for: Berberine attenuates diabetic kidney injury via FOXO1/SP1-associated regulation of renal SGLT-2 expression
Source: Chin Med. 2026 Sep 29;21:225. doi: 10.1186/s13020-026-01533-7 (PMC13621674; doi:10.1186/s13020-026-01533-7)
Supplement: Supplementary file 1 [file 13020_2026_1533_MOESM1_ESM.docx]

A lentiviral vector for human FoxO1 overexpression was purchased from Hanbio Biotechnology (Shanghai, China). The vector was labeled with ZsGreen-PURO, contained a 3×Flag tag, and was driven by the CMV promoter.

**Figure 1 Western bolt**

**Figure 2 Liver H&E staining**

**Figure 3 Preliminary experiment for determining the high-glucose concentration**
